# Multispectral imaging reveals biblical-period inscription unnoticed for half a century

**DOI:** 10.1371/journal.pone.0178400

**Published:** 2017-06-14

**Authors:** Shira Faigenbaum-Golovin, Anat Mendel-Geberovich, Arie Shaus, Barak Sober, Michael Cordonsky, David Levin, Murray Moinester, Benjamin Sass, Eli Turkel, Eli Piasetzky, Israel Finkelstein

**Affiliations:** 1 Department of Applied Mathematics, Sackler Faculty of Exact Sciences, Tel Aviv University, Tel Aviv, Israel; 2 Jacob M. Alkow Department of Archaeology and Ancient Near Eastern Civilizations, Lester and Sally Entin Faculty of Humanities, Tel Aviv University, Tel Aviv, Israel; 3 School of Physics and Astronomy, Sackler Faculty of Exact Sciences, Tel Aviv University, Tel Aviv, Israel; Seoul National University College of Medicine, REPUBLIC OF KOREA

## Abstract

Most surviving biblical period Hebrew inscriptions are ostraca—ink-on-clay texts. They are poorly preserved and once unearthed, fade rapidly. Therefore, proper and timely documentation of ostraca is essential. Here we show a striking example of a hitherto invisible text on the back side of an ostracon revealed via multispectral imaging. This ostracon, found at the desert fortress of Arad and dated to ca. 600 BCE (the eve of Judah’s destruction by Nebuchadnezzar), has been on display for half a century. Its front side has been thoroughly studied, while its back side was considered blank. Our research revealed three lines of text on the supposedly blank side and four "new" lines on the front side. Our results demonstrate the need for multispectral image acquisition for both sides of all ancient ink ostraca. Moreover, in certain cases we recommend employing multispectral techniques for screening newly unearthed ceramic potsherds prior to disposal.

## Introduction

**Fig 1:**
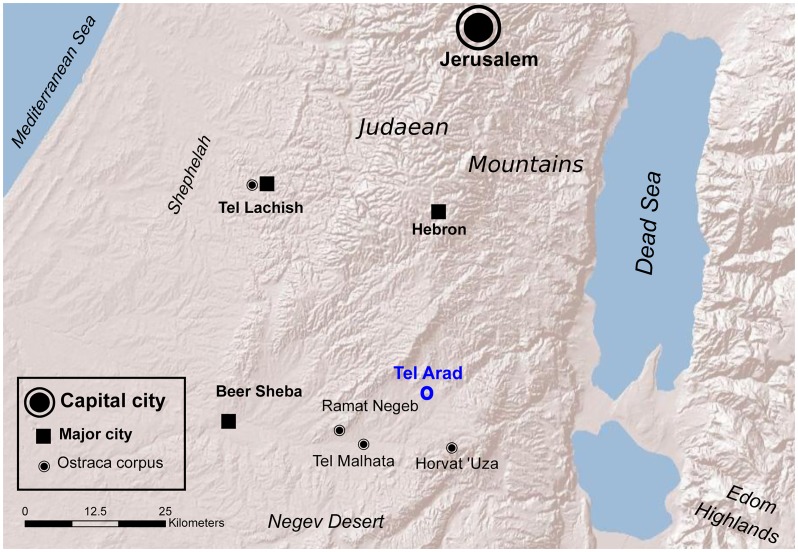
The Kingdom of Judah. Main towns in Judah and sites with major ostraca findings.

The final years of the Kingdom of Judah, ending with the destruction of Jerusalem by the Babylonian king Nebuchadnezzar in 586 BCE, are characterized by strong literary activity [1]. The more significant texts of this era were probably written on papyri, which did not survive due to the local humid climate. The majority of existing texts unearthed in archaeological excavations are Hebrew ink inscriptions written on ceramic potsherds (ostraca). They generally deal with mundane issues such as orders related to troop movement and shipment of provisions, records of ownership, and name lists. The foremost corpora of ostraca were found at the sites of Tel Arad [2] and Ḥorvat ‘Uza [3] in the southern arid zone and Tel Lachish [4] in the Shephelah (the western lowland) of Judah (Fig 1).

Ostraca are affected by post-depositional processes and hence their preservation is typically poor. They are frequently effaced, blurred, and stained, with often barely perceptible traces of ink. Furthermore, commonly, following excavation, the ink of the ostraca fades more rapidly than it had in the ground. From our observations, even over a relatively short period of 20 years after having been discovered, the ink on an ostracon might deteriorate noticeably [5]. Hence proper and timely documentation of ostraca after being unearthed is of paramount importance.

Modern documentation of inscriptions is usually based on their digital images. Legible images can aid epigraphers (specialists in ancient scripts and texts) in deciphering these challenging texts. As ostraca are usually quite small (approximately 5 to 10 cm across), characteristically containing only 20–50 characters, the identification of a single sign may affect the understanding and interpretation of a word, sentence, and even of the entire inscription.

Typically, a newly discovered ostracon is imaged using a standard digital camera. In some cases, infrared (IR) imaging is used, allegedly improving the legibility of the inscription [6,7]. In a recent study [8], we showed the advantage of multispectral (MS) imaging over standard as well as IR photography. This research suggested a simple procedure for acquiring the most legible MS image out of a group of images taken at different wavelengths. Our experiments demonstrated that although the optimal imaging wavelength of each ostracon may differ, it nevertheless belongs in the range between 550 and 950 nm. In addition, we showed that in order to capture the most favorable images of a wide assortment of ostraca, ten different band pass filters, partitioning the 550–950 nm range, are sufficient. Based on these conclusions, a low-cost MS acquisition system was constructed and compared to a more sophisticated and costly MS imaging device. The potential for legibility improvement was found to be comparable in these two systems. For additional details regarding our acquisition mechanism, see the Materials and methods section below.

The MS imaging technique was applied to several ostraca from various locations and periods, and has shown significant potential for text legibility improvement. Among these ostraca are inscriptions from the end days of the Kingdom of Judah (ca. 600 BCE) found at several sites in the arid, southern Beer Sheba Valley [5,9], as well as in Jerusalem, the capital of Judah [10]. The reading of a much earlier, Late Bronze Age inscription (12^th^ century BCE) from Qubur el-Walaydah in southern Israel, written in Hieratic script (a cursive Egyptian writing system), also benefited from our technique [11]. These examples demonstrate that at least some of the ostraca have ink traces invisible to the naked eye that are detectable by MS photography. They also indicate that in certain cases MS imaging can provide good results even decades after excavation despite overall ink deterioration.

The study reported here is the first attempt to acquire MS images of an ostracon that belongs to the important corpus of epigraphic finds from Tel Arad. The fortress of Arad is located in arid southern Judah, on the border of the kingdom with Edom. The Arad corpus, unearthed in the 1960s [2], includes about 100 Hebrew inscriptions. The inscriptions contain commands regarding supply of commodities (wine, oil, and flour) to military units and movement of troops, set against the background of the stormy events in the final years before the fall of Judah. They include orders that came to the fortress of Arad from higher echelons in the Judahite military command system, as well as correspondence with neighboring forts. Most of the provision orders were found on the floor of a single room. They are addressed to a person named Elyashiv—the quartermaster in the fortress (for additional information regarding Elyashiv’s position within the Judahite army’s chain of command, see [1]). The corpus provides important extra-biblical evidence for Judah’s final tumultuous years, as well as a vital source for linguistic studies of ancient Hebrew and other Semitic languages [12–14].

In this paper we focus on one of the inscriptions, the epigraphically well-studied Ostracon No. 16 (Israel Antiquities Authority number: 1967–990), dated to ca. 600 BCE. This is a letter sent to Elyashiv from one Ḥananyahu (possibly a quartermaster in Beer Sheba, and thus Elyashiv’s peer [1]), mentioning transfer of silver (used as a currency). Excavated in 1965 and displayed in the Israel Museum, the front side (*recto*) of this ostracon was thoroughly studied by experts over the years (e.g. [12–18]). Despite half a century of scrupulous examinations of this inscription, its back side (*verso*) was considered void of any ink traces. Contrary to this common belief, our MS imaging of the *verso* revealed three clear lines of text, exposed here for the first time. Additionally, our MS imaging of the *recto* added four previously invisible lines, and in the discernible part substantially changed previous readings.

## Materials and methods

A previous experimental study conducted by our team [8] demonstrated that: (a) the optimal imaging wavelength for ostraca lies in the range of 550–950 nm and (b) ten different bandpass filters are sufficient for capturing the most favorable image. During this research, attempts at combining various multispectral channels (e.g. PCA) were performed. The experimentations revealed that linear combinations of several bands result in only marginal improvement, at best. On the other hand, locating the best possible multispectral image, taking into account all its possible grayscale transformations, was found to be more beneficial. For details see S1 Appendix.

In the current research, we used a standard digital camera that is sensitive to the visual spectrum (i.e., 400–700 nm), with its internal infra-red (IR) cut filter removed and replaced with transparent glass, in order to enhance the camera sensitivity in IR wavelengths (i.e., up to 1000 nm). Thereafter, the spectrum was sliced into ten channels utilizing commercial external bandpass filters. Using this system, we produced spectral cubes of ten images for each side of the ostracon. We selected the best images (taken at 830 nm for the *recto* and 890 nm for the *verso*) based on the potential contrast algorithm that we developed [8,19]. In order to improve the legibility of the images, we performed contrast and brightness manipulations via the freely available ImageJ and IrfanView software applications.

**Fig 2:**
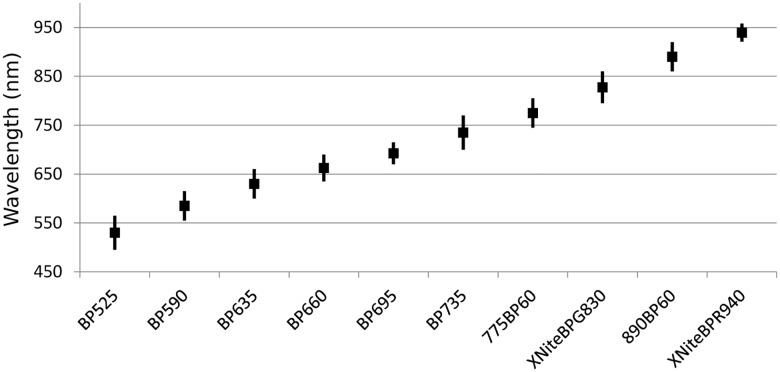
Band-pass filters coverage. Off the shelf filters covering the complete range of 550–950 nm, each with a window of approximately 60 nm.

The specifications of the current experiment were as follows. Camera details: We used a modified Canon SLR 450D digital camera, and a Tamron SP AF90mm F/2.8 Di 1:1 Macro lens. The internal Canon IR cut filter was removed by Lifepixel [20] and replaced with transparent glass having the same refractive index. The converted camera has useful quantum efficiency from about 400 nm up to 1000 nm [21,22], which follows from the characteristics of the CMOS imaging chip sensor. Ten "off the shelf" bandpass filters were utilized. Five filters were produced by MidOpt [23], with transmission centers at 525, 590, 635, 660, 695 and 735 nm. Two filters were manufactured by Omega [24], with transmission centers at 775 and 890 nm. The remaining two filters were produced by Maxmax [25], with transmission centers at 830 and 940 nm. The filters’ bandwidths are 40 up to 70 nm. Fig 2 shows the coverage of the range 550–950 nm, using these filters. The artifact was imaged in a dark room with interchanging filters from a distance of ca. 50 cm, with two light bulbs (GE halogen 80 watt PAR38 floodlight [26]) located at 45° from both sides of the ostracon with respect to the optical axis. Due to a slight de-focusing after each filter switch, a re-focus procedure is performed at each filter replacement.

The archaeological specimen discussed in this study is the Arad 16 ostracon, unearthed by Prof. Yohanan Aharoni in 1965. The ostracon is dated to ca. 600 BCE. According to the original excavation report [2], it was found in Stratum VI, Square L9, Locus 637, along with ostraca 1–15,17–18 (the so called “Elyashiv archive”, named after the main addressee of the inscriptions). Its dimensions are 64×90 mm, with 6 mm thickness. The sherd is a broken part of a storage jar.

Permits for imaging, research and publication of the Arad 16 ostracon (Israel Antiquities Authority number: 1967–990); located in the Israel Museum, Jerusalem (the specimen number is the same as the Israel Antiquities Authority’s one), were obtained from the Israel Antiquities Authority. All necessary permits for the research, as well as the publication of the specimen were obtained for the described study, which complied with all relevant regulations.

## Results

**Fig 3:**
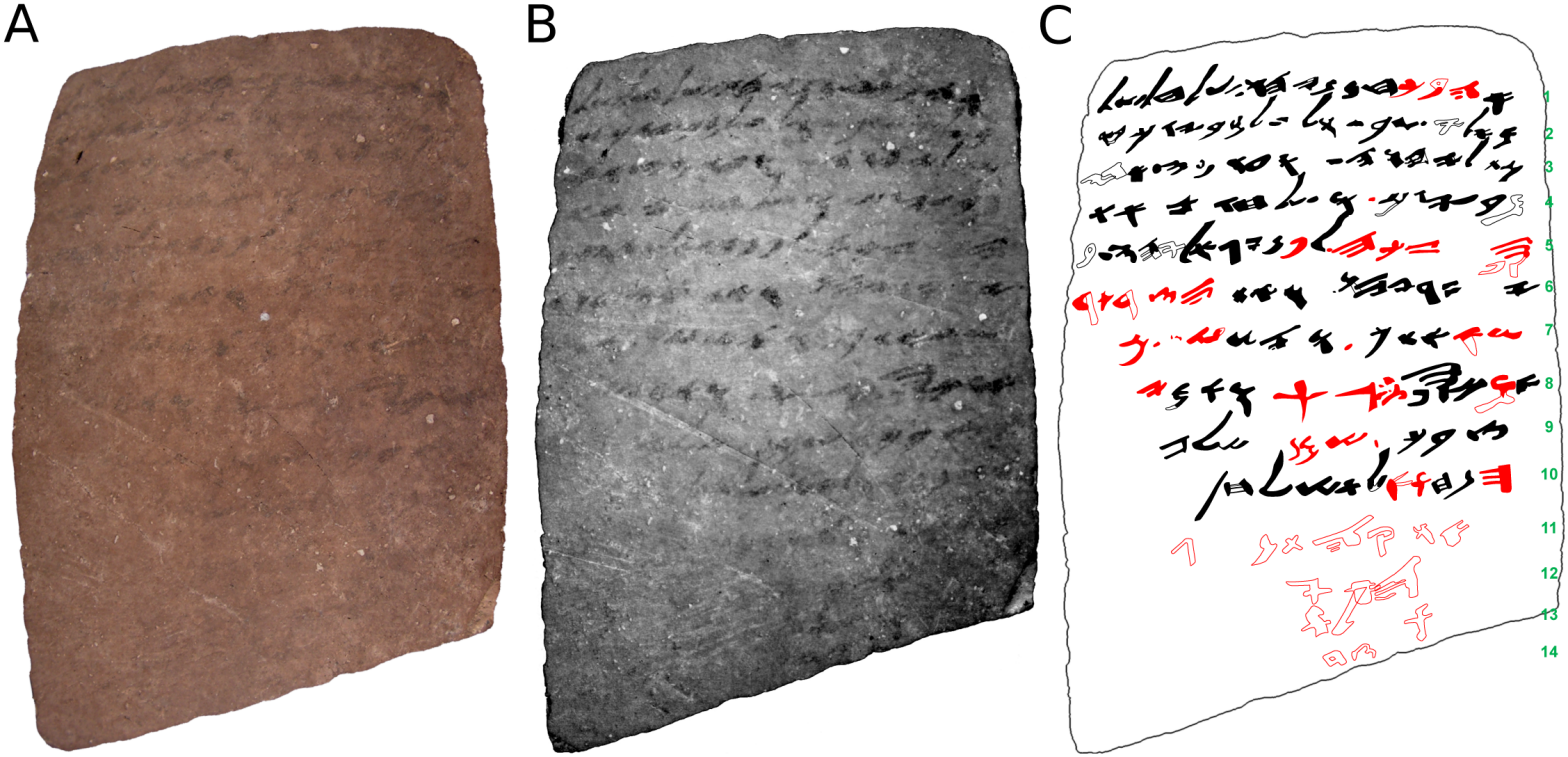
The *recto* of Arad ostracon No. 16. (A) color (RGB) image; (B) MS image corresponding to 830 nm; (C) manual drawing (facsimile) of the proposed reading. In **red**: our alterations and additions with respect to the original publication (*editio princeps*) [2]. Hollow shapes represent conjectured characters.

**Fig 4:**
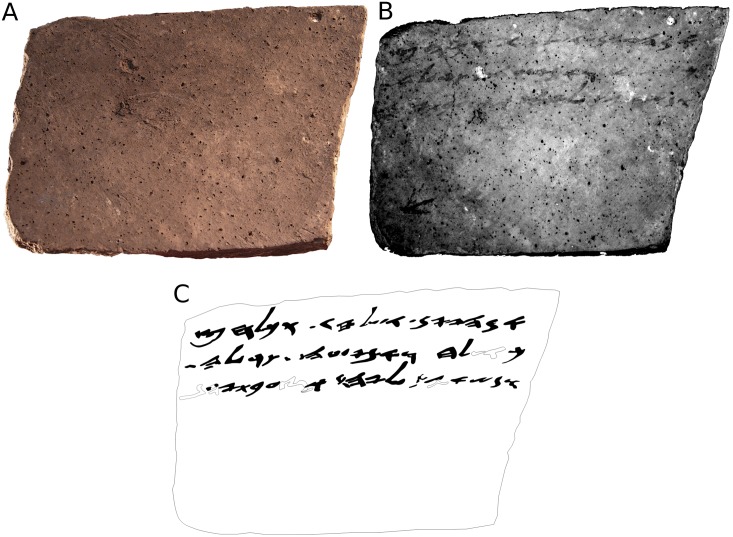
The *verso* of Arad ostracon No. 16. (A) color (RGB) image; (B) MS image corresponding to 890 nm; (C) manual drawing (facsimile) of the proposed reading. Hollow shapes represent conjectured characters.

Originally, our sole target for MS imaging was the *recto* of Arad Ostracon 16, with uneven preservation of writing across its surface. However, while handling the *recto*, a suspicion arose regarding a possible existence of writing on the *verso*. A presence of a *verso* text could provide a continuation of the inscription on the *recto*, the surface of which was entirely covered by writing (as we can learn from the new MS image in Fig 3b). The MS imaging procedure confirmed the existence of text on the *verso* (see Fig 4b). Indeed, three lines of writing can now be seen for the first time since its excavation half a century ago and more than two and a half millennia after it was first inscribed.

The procedure was similar for both sides of the ostracon. Using our MS device [8], we produced a spectral cube of ten images for each side. The most legible images were selected based on an algorithm described in [8,19]. Additionally, we performed contrast and brightness manipulations via the freely available ImageJ and IrfanView software applications. Subsequently, drawings (facsimiles) of the inscriptions were created manually.

**Fig 5:**
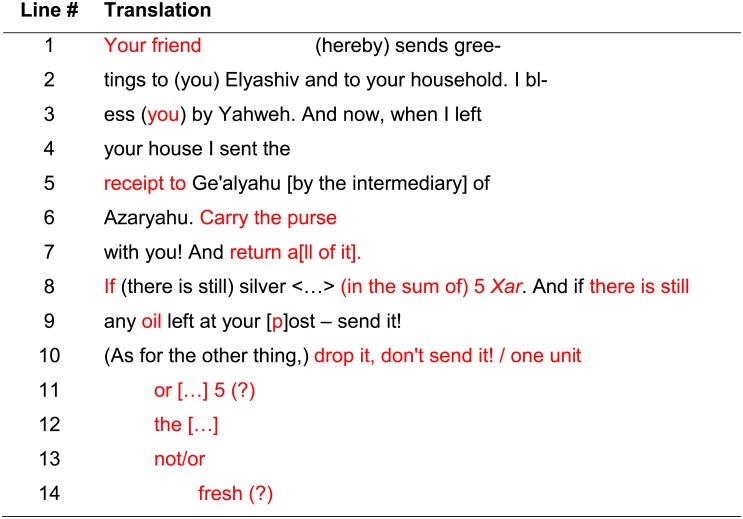
Renewed reading of Arad 16 *recto*. In **red**: our alterations and additions with respect to the original publication (*editio princeps*) [2].

The most legible MS images of the *recto* corresponded to the wavelength of 830 nm. In the original publication (*editio princeps*), the rendering of lines 1–3 was considered to be certain, with the partial reading of lines 4–10 considered as tentative [2]; later scholars noted the existence of additional lines but were unable to decipher them [12–18]. The chosen MS image enhanced the legibility of the inscription. This enabled us to alter the decipherment of lines 1, 5 and 6, to read lines 7–10 with certainty, and to propose an incomplete reading of the previously unseen lines 11–14. For color (RGB) and MS images of the *recto*, as well as the facsimile of the proposed reading, see Fig 3; the translation of the *recto* is presented in Fig 5.

**Fig 6:**
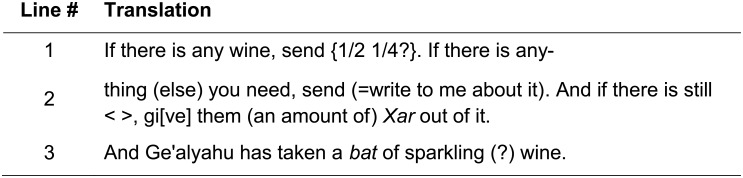
First reading of Arad 16 *verso*.

In the *verso*, the most legible image corresponded to the wavelength of 890 nm. A comparison between the color and MS images of the *verso* is presented in Fig 4a and 4b, respectively. While no text is discernible on Fig 4a, three lines of writing are apparent on Fig 4b (the fact that the two sides of the ostracon had varying levels of legibility can be explained by post-depositional processes, which may have affected them differently). The facsimile of the inscription depicts a reasonably certain reading; it is presented in Fig 4c. The translation of the *verso* is presented in Fig 6.

The new reading of the *recto* of Arad 16 (see Fig 5) has added about 45 new characters. There are almost 20 words on the *recto* with a changed reading (half of the total!). The text details an exchange of supplies and silver between Elyashiv, the quartermaster of the Arad fortress and Ḥananyahu, possibly his peer at Beer Sheba, located one day's walk (ca. 25 km) to the west. Elyashiv and Ḥananyahu seem to be on friendly terms, with the letter possibly continuing an earlier personal communication. The letter begins with an affectionate salutation (“Your friend Ḥananyahu [hereby] sends greetings to [you] Elyashiv and to your household”) and continues with a blessing from God (“I bless [you] by Yahweh”). This is followed by the mention of a receipt sent by Ḥananyahu (“when I left your house I sent the receipt to Ge'alyahu”). Requests regarding a certain purse and an amount of silver (“in the sum of 5 *Xar*”), as well as oil (“if there is still any oil left at your [p]ost—send it”) are made, along with a call to avoid sending a certain commodity, the name of which is indiscernible (“[…] drop it, don't send it!“). The last part of the *recto* contains some decipherable signs and letters, which, however, do not amount to a coherent text.

The *verso* is seen and deciphered by us here for the first time (for our reading of the text see Fig 6). The text bears more than 50 characters, creating 17 new words. It begins with a request for wine (“If there is any wine, send [quantity]”), as well as a guarantee for assistance if the addressee has any requests of his own (“If there is anything [else] you need, send [= write to me about it]”). The letter concludes with a request for the provision of a certain commodity to an unnamed person, and a note regarding a *bat* (an ancient measure of liquids, see [27]) of wine carried by a man named Ge'alyahu.

The *verso* seems to be a continuation of the *recto*. Supporting evidence for this hypothesis include: a full utilization of the *recto* surface for writing; an absence of an opening or greeting formula on the *verso*–a mainstay of ancient Hebrew correspondence; the similar topics of the two texts (details regarding the provision of supplies); and the probable mention of the same person, Ge'alyahu, on both sides. Therefore, it seems that when the entire surface of the *recto* was exhausted, the writer continued the same text on the *verso*: the ostracon was turned over and then turned clockwise by 90° in order to write the additional three lines on the *verso*. The rotation has a simple geometrical rationale. The shape of the ostracon was trapezoidal rather than rectangular (see Figs 3 and 4). Upon turning over, an adjustment to writing direction was required, and the writer rotated the ostracon by 90°.

## Discussion

Ostraca from various sites within the borders of the ancient Kingdom of Judah provide us with a unique glimpse at the daily activity of its administrative systems on the eve of the Babylonian destruction in 586 BCE. Regrettably, these inscriptions, several hundred in total, are almost the sole surviving textual evidence from this period. Hence, their documentation, accompanied by the most accurate and complete transliteration, is of utmost importance for the fields of Archaeology, Biblical Studies and Northwest Semitic Philology. Indeed, the case of Ostracon No. 16 from Arad demonstrates the importance of our MS imaging technique for the study of ancient ostraca in general, and biblical period ostraca from the Kingdom of Judah in particular.

Preliminary inspection of MS images of other First Temple period Hebrew ostraca demonstrates that the results for Ostracon No. 16 are not unique. Table 1 provides information regarding the expected reading improvements in other inscriptions, according to the amount of changed or new characters (including ostraca published in [3,28,29]). Some of these amendments necessitate substantial revisions to the currently accepted readings.

**Table 1:** Estimation of MS-induced reading improvements within First Temple period Hebrew ostraca.

Number of new/changed characters	Inscription
**1–5**	Arad 8, Arad 12B
**6–10**	Arad 3B, Arad 11, Arad 40, Arad 76, Ḥorvat ‘Uza 17,
**11–30**	Arad 12A, Arad 21, Arad 28, Arad 49, City of David 5, Ramat Bet Shemesh 1, Ramat Bet Shemesh 2, Ḥorvat ‘Uza 1
**31+**	Arad 24A

These examples demonstrate the need for acquiring proper MS images of both sides of all ostraca unearthed since the early days of biblical archaeology. This is an urgent task due to the fading of the ostraca's ink following their excavation. Although MS imaging can occasionally provide legibility improvement even decades after the exposure of the ostraca (e.g. [10]), undoubtedly results would have been far superior and more complete had MS imaging been done prior to the ink deterioration process. This means that MS imaging must be carried out on the two sides of every newly-excavated ostracon immediately upon discovery. Needless to say, our recommendation for proper and timely documentation also applies to other ink inscriptions on clay from various periods, languages, writing systems, and localities.

The previously invisible inscription on the *verso* of Arad 16 presents a particularly striking case with broad implications on excavation methodology. Modern excavations tend to dispose of most collected ceramic body-sherds. To differ from rims, handles, and bases of vessels, it was exactly those pieces which were selected in antiquity for writing. Disposal of body-sherds may therefore result in a potential loss of inscriptions that have mostly faded but can be salvaged by MS imaging procedures. We therefore recommend employing MS techniques for at least random screening of sherds at sites suspected of concealing written materials. Although this may entail a certain complication in excavation procedures, the prospective gains for a better understanding of the past make the effort worthwhile.

## Supporting information

S1 Appendix Ineffectiveness of applying PCA to multispectral images of Iron Age ostraca. (PDF)
