# Supplementary material for: Multispectral imaging reveals biblical-period inscription unnoticed for half a century
Source: PLoS One. 2017 Jun 14;12(6):e0178400. doi: 10.1371/journal.pone.0178400 (PMC5470672; doi:10.1371/journal.pone.0178400)
Supplement: S1 Appendix — (PDF) [file pone.0178400.s001.pdf]

S1 Appendix: Ineffectiveness of Applying PCA to Multispectral Images of Iron Age Ostraca

Our current approach to dealing with multispectral imagery of ostraca is as follows:

1. Image a particular ostracon via standard digital camera, with its internal IR cut filter replaced with transparent glass, enhancing its sensitivity up to 1000 nm. The spectrum is sliced into ten channels utilizing commercial external bandpass filters. The output is a spectral cube of ten images.
2. The best image out of the spectral cube is selected based on the potential contrast algorithm that we developed [8,19].

It can be claimed, that combining the images, e.g. via the prominent Principal Component Analysis (PCA), rather than selecting a particular one (Step 2), may lead to better inscription's legibility. However, PCA restricts us to perfectly aligned images, while due to the nature of our current MS system (i.e. changing external bandpass filters), minor misalignments of the images may exist. These might cause difficulties with the application of PCA, even if pre-processing registration steps are applied.

This impediment was avoided in our initial large-scale experimental study [8], conducted on 33 Iron Age black ink ostraca. Among other experiments conducted in this research, we tested the PCA approach in a controlled manner on perfectly aligned images. Combining the cube channels using a PCA, was found to be ineffective. For each tested ostracon, the experiment consisted of the following steps:

1. The inscription was imaged utilizing a commercial multispectral imager (CRI Nuance VariSpec SNIR-10), comprising a short and near infrared liquid crystal tunable filter (LCTF). The output of this device was a spectral cube of 51 fully registered images.
2. Each image in this cube was assessed via the potential contrast algorithm (e.g. Table S1 and Fig S2). In addition, the resulting 51 principal component images of a relevant region of interest were also assessed in the same manner (e.g. Table S2 and Fig S3), with 4 different configurations:
    A. PCA based on the raw data
    B. PCA based on standardized data, with mean=0 and std=1
    C. PCA based on data with mean=0
    D. PCA based on data with std=1

The results of this experiment demonstrated that the quality of the best image out of the cube was better than the quality of all the principal component images, in all

configurations. In fact, typically, almost all the cube images had better quality than the principal component images.

Below we present an example of such an experiment on Ḥorvat Radum ostracon #1 ([3]; see Fig S1). In Table S1, potential contrast quality scores for its cube images are presented. The best score (marked in red) is 225.19, achieved for central wavelength of 600 nm. Our past observations [8] demonstrated that cube channels with scores above 95% of the optimal one, provide comparable legibility. In the current case, all the channels in the range 570-720 nm have passed that mark. Examples of some of these images can be seen in Fig S2.

Table S2 presents potential contrast quality score for the principal component images of the same ostracon, for all PCA configurations (A-D). The best result is 221.50, corresponding to the first principal component of configuration A. The quality of the images corresponding to principal components after the first few, deteriorates rapidly for all configurations. Representative images of the principal components in different configurations can be seen on Fig S3.

The results indicated that our current method of selecting the best cube image is preferable to that of images' combination via various PCA configurations. Upon in-depth investigation, it seems that the best principal component result represents an approximate averaging of the cube channels. In other words, the cube images are strongly correlated and difficult to discriminate. The lion share of the principal components (i.e. "distinguishing" information) represents noise emanating from the highly irregular background medium.

The empirical results demonstrated that combining the channels via PCA is not preferable to selecting the most contrasted cube image. Therefore, obtaining perfectly aligned cube images is not essential. This observation allowed us to settle on a low-budget multispectral imaging system. The device is suitable for the achievement of the goals of our project, among which is a legibility improvement of ostraca, as demonstrated in the current article.

Table S1. Potential contrast quality score for the spectral cube images of Ḥorvat Radum ostracon #1. The best result is highlighted in red.

Center Wavelength (nm)	Potential Contrast Score
450	215.55
460	208.68
470	208.96
480	205.18
490	202.42
500	197.47
510	194.37
520	192.72
530	191.24
540	192.18
550	198.68
560	205.96
570	215.12
580	222.62
590	225.14
**600**	**225.19**
610	223.82
620	223.84
630	221.60
640	221.58
650	220.87
660	219.81
670	219.17
680	217.52
690	217.15
700	215.94
710	214.81
720	214.45
730	213.18
740	212.32
750	212.21
760	212.50
770	211.05
780	211.57
790	212.45
800	213.30
810	213.63
820	212.69
830	214.06
840	213.94
850	212.49
860	209.78
870	209.12
880	207.15
890	204.57
900	201.28
910	198.16
920	193.00
930	187.21
940	181.19
950	176.03

Table S2. Potential contrast quality score for the principal component images of Ḥorvat Radum ostracon #1. The best result is highlighted in red.

Principal Component	Potential Contrast Score			
	PCA based on the raw data	PCA with mean=0 and std=1	PCA with mean=0	PCA with std=1
**1**	**221.50**	87.94	140.83	114.08
2	142.35	**151.37**	**151.13**	**142.72**
3	111.72	41.64	32.46	131.06
4	36.33	46.80	35.88	47.51
5	24.01	28.93	24.05	29.92
6	37.73	29.44	36.13	18.38
7	24.03	35.91	42.79	23.65
8	62.38	64.25	36.40	70.43
9	21.75	21.67	30.21	22.73
10	21.48	24.95	24.99	23.00
11	24.34	21.60	25.64	21.53
12	22.07	18.87	22.49	20.51
13	27.35	22.41	22.18	20.73
14	23.12	18.69	23.11	21.85
15	25.33	24.26	24.20	23.26
16	22.48	21.68	21.86	20.68
17	23.65	18.47	25.02	19.98
18	23.17	20.90	23.74	20.54
19	22.47	22.01	25.25	20.99
20	23.09	19.07	26.21	20.14
21	23.60	21.49	23.86	21.77
22	22.61	21.01	20.51	22.99
23	23.88	20.59	23.72	19.88
24	23.12	18.05	25.12	18.14
25	24.53	18.96	23.40	18.87
26	27.75	21.92	26.64	21.92
27	23.78	18.73	24.39	19.27
28	31.35	18.78	26.73	19.61
29	27.41	16.62	29.73	16.77
30	26.76	21.10	26.59	21.28
31	29.14	18.82	27.85	18.09
32	25.07	19.89	23.70	20.45
33	27.57	18.00	25.41	18.58
34	27.54	16.72	29.36	17.99
35	29.50	15.88	28.45	19.52
36	23.14	22.74	24.63	17.72
37	28.26	14.50	26.26	15.27
38	25.79	19.45	26.42	16.31
39	27.16	20.63	26.65	20.70
40	27.63	17.82	29.17	13.61
41	27.05	19.99	22.44	13.99
42	23.38	16.83	26.96	16.98
43	27.14	17.41	25.64	16.21
44	25.15	17.20	26.33	17.04
45	25.06	16.14	25.93	17.61
46	26.12	17.10	26.28	16.88
47	23.41	17.02	24.20	16.99
48	27.97	18.53	25.03	18.98
49	25.51	16.85	24.03	18.39
50	24.03	20.42	23.57	20.69
51	23.68	17.02	22.15	19.97

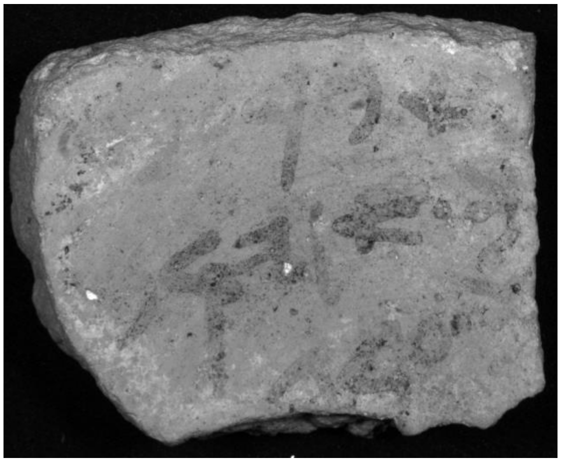

Fig S1. Ḥorvat Radum ostracon #1. Image of the ostracon at λ=600 nm.

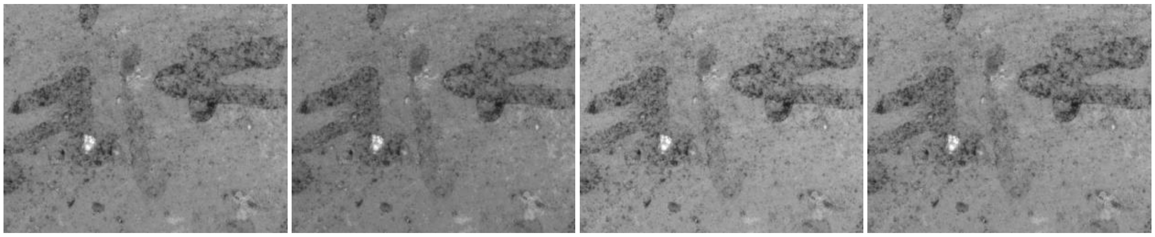

Fig S2. Ḥorvat Radum ostracon #1, zoom-in on a region of interest - the top-4 scoring image cube channels. From left to right, image corresponding to: λ=600 nm (score=225.19); λ=590 nm (score=225.14); λ=620 nm (score=223.84); λ=610 nm (score=223.82). No post-processing of the images was performed.

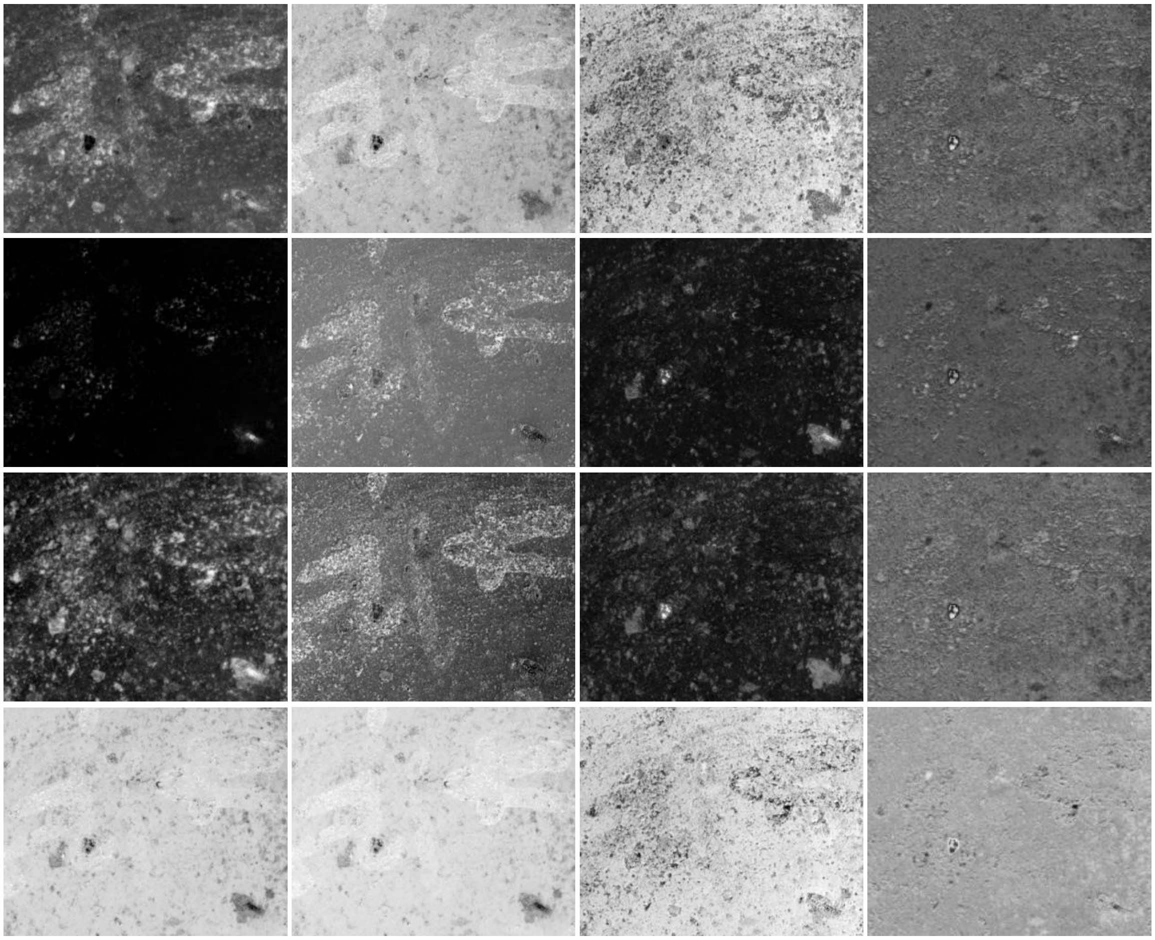

Fig S3. Ḥorvat Radum ostracon #1, zoom-in on a region of interest of the first 4 principal component images, in all configurations. From top to bottom PCA configurations A-D, from left to right – principal components 1-4; for scores see Table S2.
